# Hydrophobic Phenolic/Silica Hybrid Aerogels for Thermal Insulation: Effect of Methyl Modification Method

**DOI:** 10.3390/gels12010004

**Published:** 2025-12-20

**Authors:** Mengcheng Nie, Yong Kong, Zhixin Wang, Fuhao Xu, Jiantao Zhou, Xiaodong Shen

**Affiliations:** 1 College of Materials Science and Engineering, Nanjing Tech University, Nanjing 211816, China; 2 Jiangsu Collaborative Innovation Center for Advanced Inorganic Function Composites, Nanjing 211816, China

**Keywords:** phenolic/silica aerogel, hydrophobic, weather resistance, thermal conductivity, stability

## Abstract

Hydrophobic phenolic/silica hybrid aerogels were synthesized via different methyl modification methods including in situ polymerization (RA-IS), surface grafting (RA-SG), and vapor deposition (RA-VD). All the methods achieved good hydrophobicity, with a water contact angle around 140°, and the hydrophobic mechanisms were clarified. RA-IS possesses the highest specific surface area and nanopore volume, and the lowest bulk density. Therefore, it exhibits much lower thermal conductivity (32.2 mW·m^−1^·K^−1^) at 25 °C than RA-SG, RA-VD and other reported phenolic/silica hybrid aerogels. The compression strength (3.3 MPa) and Young’s modulus (19.2 MPa) of RA-IS are higher than those of its state-of-the-art counterparts. The methyl groups in RA-IS are linked in the matrix by a covalent bond, leading to excellent weather resistance under thermal, hygrothermal, and ultraviolet aging conditions. The methyl species in RA-SG and RA-VD are loaded on the surface via a covalent bond and physical adsorption, exhibiting poor weather resistance. RA-IS is incombustible and its microstructure is stable on an alcohol flame. This study provides new insights into the hydrophobicity of phenolic/silica hybrid aerogels, and offers significant guidance for developing aerogels with high strength, hydrophobicity, flame resistance, weather resistance, and insulation performance for building insulation.

## 1. Introduction

Recently, excessive emissions of greenhouse gases have generated an increasing atmosphere temperature, owing to the overuse of fossil fuels [1,2,3]. Global warming has become one of the most pressing challenges confronting human society [4,5,6]. The energy consumption of buildings accounts for ~50% of the total energy consumption according to the China Association of Building Energy Efficiency. The use of high-performance thermal insulation materials is an effective measure to reduce carbon emissions of buildings. Silica aerogels have low thermal conductivity and high temperature resistance, and they have been widely used in many fields such as aerospace, the petrochemical industry, and electric vehicles. However, their poor mechanical properties restrict their application in building insulation [7,8,9,10,11,12,13,14,15,16,17].

Phenolic aerogels exhibit much better mechanical properties than silica aerogels [18,19,20]. Moreover, their thermal conductivities are as low as those of silica aerogels [11,21,22]. However, phenolic aerogels exhibit poor thermal stability and fire safety for building insulation. Introducing inorganic components is an efficient method to enhance the thermal stability of phenolic aerogels [23,24]. Generally, the introduction of inorganic components into phenolic aerogels is achieved by the dopant incorporation of powdery silica or the in situ co-polymerization of precursors of silica. It is difficult to achieve a uniform distribution in silica for powder-doping. Hence, phenolic/silica hybrid aerogels have been extensively developed via the in situ co-polymerization of the precursors of phenolic and silica. Typically, phenolic resin, resorcinol (R), and formaldehyde (F) are used as the precursors of phenolic-based aerogels. Tetraethyl orthosilicate (TEOS) and 3-(aminopropyl)triethoxysilane (APTES) are mostly used as precursors of silica aerogels [25,26,27]. For instance, Pekala et al., first reported resorcinol–formaldehyde (RF) aerogels prepared by the base-catalyzed polycondensation of resorcinol and formaldehyde followed by supercritical drying, which showed that its density was as low as 0.03–0.1 g/cm^−3^ [28]. Lu et al. subsequently demonstrated that the thermal conductivity of resorcinol–formaldehyde (RF) aerogels is comparable to that of silica aerogels, reaching 0.012 W·m^−1^·K^−1^ at ρ = 0.157 g·cm^−3^ [29]. More recent work has optimized RF synthesis conditions and crosslinking strategies to tune the pore structure and compressive behavior of RF aerogels from hard and brittle to highly flexible. The density of the covering aerogel ranges from 0.08 to 0.3 g·cm^−3^, and the compressive modulus ranges from 0.12 to 28 MPa [30,31]. In addition, RF/silica hybrid aerogels prepared in one pot by the co-gelation of RF with TEOS/APTES exhibit a more uniform organic–inorganic network, resulting in a specific surface area of 416 m^2^·g^−1^, comparable to that of pure RF aerogels [25].

These studies indicate that phenolic and RF-based aerogels, especially RF/silica hybrids, combine the ultra-low thermal conductivity and high porosity typical of silica aerogels with much higher compressive strength, better dimensional stability, and, after carbonization, high temperature resistance, which make them attractive candidates for structural thermal insulation and high-temperature applications. However, there are abundant hydrophilic hydroxyl groups on the surface of the framework of phenolic/silica hybrid aerogels, leading to their poor durability in humid environments.

So far, systematic studies on the hydrophobic modification of phenolic or phenolic/silica hybrid aerogels are still scarce. In contrast, a variety of strategies have been demonstrated to achieve hydrophobicity, including in situ polymerization, surface grafting, and vapor deposition. In situ polymerization is conducted using methyl-functional silane as co-precursors, such as methyltrimethoxysilane (MTMS) and methyltriethoxysilane (MTES) [32,33,34,35]. Surface grafting is conducted by grafting trimethylchlorosilane (TMCS), hexamethyldisilazane (HMDS), and other methyl-functional silanes onto pre-formed wet gels [36,37]. Vapor deposition of methyl-functional silanes or polysiloxanes onto silica aerogels is another effective way to generate hydrophobic or even superhydrophobic aerogels while maintaining high transparency and low thermal conductivity [38,39,40,41,42,43]. Ye et al. prepared a phenolic/silica hybrid aerogel with a water contact angle of 148° by the in situ polymerization of MTMS. However, it was synthesized via a complicated surfactant-assisted acid–base-catalyzed two-step sol–gel process [32]. Wang et al. reported a hydrophobic phenolic/silica aerogel composite with a low thermal conductivity of 0.021 W·m^−1^·K^−1^ and a water contact angle of 138° [10]. However, its long-term stability in outdoor environments is only fair, which may be attributed to the fact that the stability of methyl groups loaded by vapor deposition is poor. Therefore, comprehensive insights into the hydrophobicity of phenolic/silica hybrid aerogels are significant for developing high-performance building insulation materials.

In this work, hydrophobic phenolic/silica (RF/SiO_2_) hybrid aerogels were prepared within the same RF–APTES system using three distinct methyl modification routes (in situ polymerization, surface grafting and vapor deposition) based on a one-step sol–gel process followed by supercritical CO_2_ drying. By directly comparing these routes at a fixed composition, we clarify how the location and bonding state of methyl groups govern the microstructure, density/shrinkage, weather resistance, thermal insulation and mechanical properties of the aerogels. This side-by-side study provides new insights into the design of hydrophobic phenolic/silica hybrid aerogels and offers practical guidance for developing materials that combine low thermal conductivity, high strength and excellent long-term durability for building insulation applications.

## 2. Results and Discussion

### 2.1. Hydrophobic Conditions and Mechanisms

**Figure 1:**
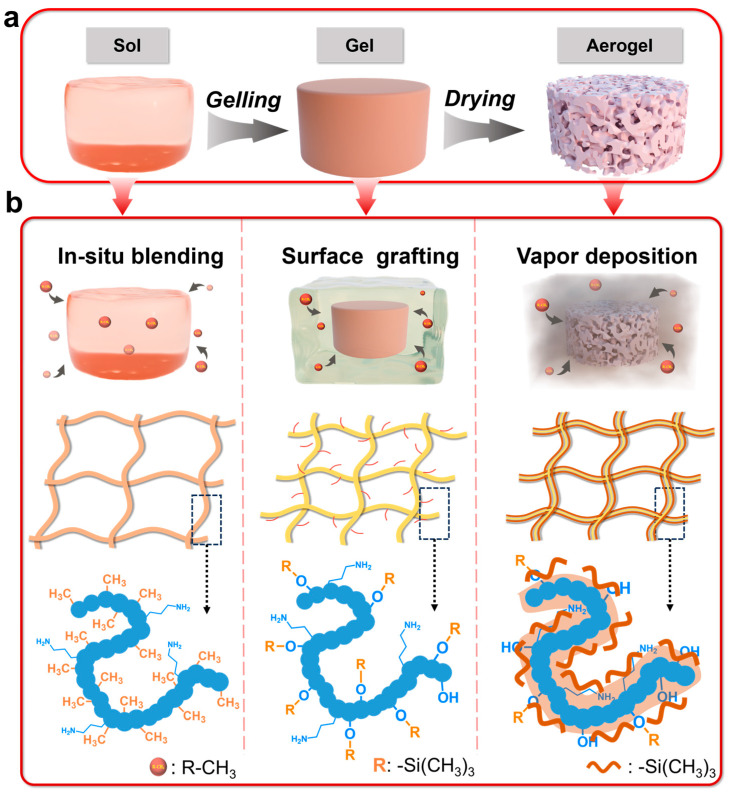
(**a**) Synthesis process of RF/SiO_2_ aerogels; (**b**) hydrophobic mechanisms of RF/SiO_2_ aerogels with different methyl modification methods.

It was found that RF/SiO_2_ aerogel can be facilely synthesized by mixing resorcinol (R), formaldehyde (F), APTES, and ethanol in a container to conduct a sol–gel reaction [44,45,46,47]. Without methyl modification, the resulting RF/SiO_2_ aerogel (denoting as RA) is hydrophilic. Figure 1a shows the sol–gel process of synthesizing hydrophobic RF/SiO_2_ aerogels. Methyl modification via in situ polymerization, surface grafting, and vapor deposition is conducted at different stages during the synthesis process of RA, and the resulting hydrophobic RF/SiO_2_ aerogels are denoted as RA-IS, RA-SG, and RA-VD, respectively. The methyl modification of RA-IS is conducted by tailoring the sol, i.e., adding MTMS as co-precursor. Therefore, the -CH_3_ groups are hybridized in the matrix of RA-IS via covalent bonding (Figure 1b). Water contact angles of the RA-IS samples with different R/MTMS molar ratios are shown in Table S1. The hydrophobicity of RA-IS exhibits an obvious dependence on the R/MTMS ratio. The R/MTMS molar ratio should be as high as 1.5 to achieve hydrophobicity. Superior hydrophobicity with a water contact angle of 141° is obtained with a R/MTMS molar ratio of 2. The methyl modification of RA-SG is conducted by tailoring the gel of RA, i.e., soaking an alcogel of RA in a HMDS/ethanol solution. The surface hydroxyl groups derived from APTES offer active sites for the chemical grafting of Si-(CH_3_)_3_ species; therefore, -CH_3_ groups can be grafted on the surface of RF/SiO_2_ framework (Figure 1b). Water contact angles of the RA-SG samples with different HMDS molar fractions in the HMDS/EtOH solution are shown in Table S2. Hydrophobicity with a water contact angle of 93° is attained with a HMDS molar fraction of 5%. The water contact angle increases to 140° when the HMDS molar fraction is increased to 10%. Further increasing HMDS fraction does not lead to an increase in the water contact angle. The methyl modification of RA-VD is conducted by tailoring the aerogel, i.e., treating the RA sample in a sealed container with HMDS vapor. Aerogel networks with high porosity and specific surface areas favor the diffusion and adsorption of HMDS vapor. As a result, the dominating Si-(CH_3_)_3_ species are physically adsorbed on the RF/SiO_2_ framework, and a small number of Si-(CH_3_)_3_ species can be fixed onto the RF/SiO_2_ framework via chemical grafting (Figure 1b). Water contact angles of the RA-VD samples with different modification environments are shown in Table S3. To achieve the hydrophobicity as well as RA-IS and RA-SG, the modification temperature should be maintained at a moderate level (60 °C), the modification time should be as long as 6 h, and the vacuum degree of the container should be as high as 0.06 MPa to ensure the sufficient diffusion of HMDS molecules.

### 2.2. Microstructure

**Figure 2:**
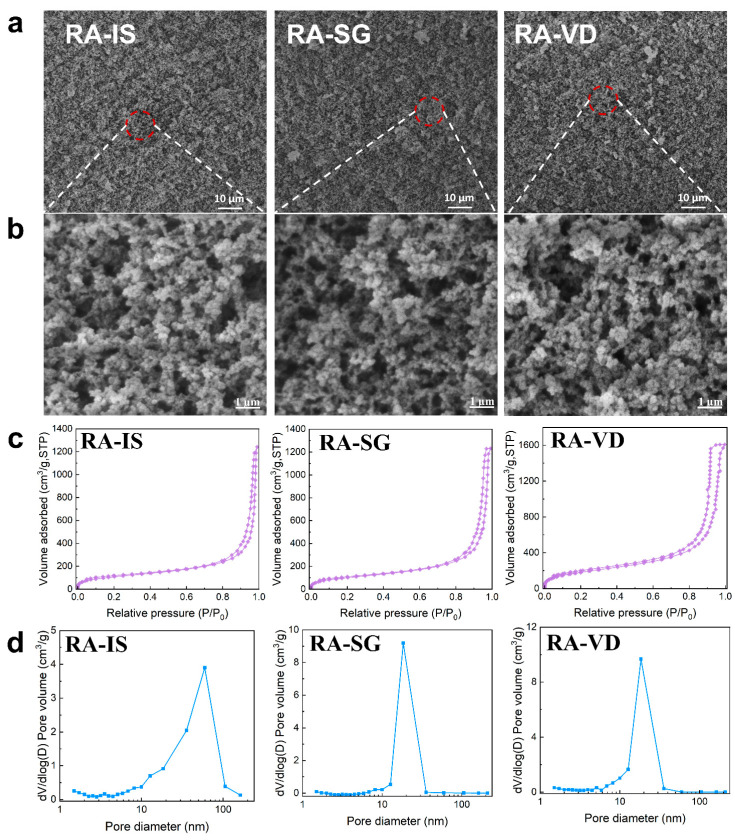
Microstructure characteristics of hydrophobic RF/SiO_2_ aerogels: (**a**,**b**) SEM images; (**c**) nitrogen adsorption–desorption isotherms; (**d**) pore size distribution curves.

Scanning electron microscope (SEM) images of RA-IS, RA-SG and RA-VD are shown in Figure 2a,b. All samples exhibit a three-dimensional porous network constructed of interconnected nanoparticles and pores. This suggests that the methyl modification method does not affect the microstructure of RF/SiO_2_ aerogels significantly. N_2_ adsorption–desorption isotherms and pore size distribution curves of the aerogels are shown in Figure 2c,d. All samples exhibit type IV isotherms with H_2_ hysteresis loops, characterizing the mesoporosity of the aerogels [48,49]. The pores of RA-IS distribute in a wide range of 5–100 nm, and most pores concentrate around 60 nm. However, a narrower pore distribution is observed for RA-SG (7–35 nm) and RA-VD (7–35 nm), and most pores of RA-SG and RA-VD concentrate around 18 nm. This is attributed to the fact that large voids can be blocked after surface grafting and the vapor deposition of HMDS species on the RF/SiO_2_ framework, generating smaller pores. In addition, the volume shrinkage can occur during aging, solvent exchange, and drying processes.

**Figure 3:**
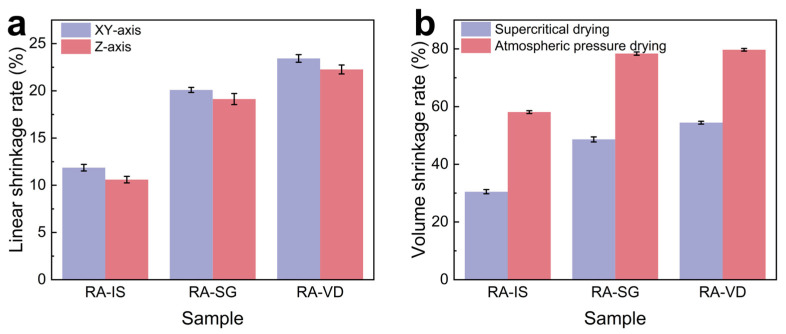
(**a**) Linear shrinkage along the XY and Z directions; (**b**) volume shrinkage.

The dimensional stability of the RF/SiO_2_ aerogels is summarized in Figure 3. As shown in Figure 3a, RA-IS exhibits the lowest linear shrinkage along both the XY and Z directions, whereas RA-SG and RA-VD show markedly higher values. This indicates that post-gel surface grafting and post-drying vapor deposition promote additional network condensation and skeleton rearrangement. The corresponding volume shrinkage rates are presented in Figure 3b. After supercritical CO_2_ drying, RA-IS again shows the smallest volume shrinkage, while RA-SG and RA-VD display increased shrinkage, consistent with their denser structures. For all three compositions, atmospheric-pressure drying leads to much larger volume shrinkage than supercritical CO_2_ drying, confirming that capillary stresses during solvent evaporation cause severe collapse of the wet gels. The densities, specific surface areas and pore volumes are shown in Table 1. With the same theoretical density, the bulk density of RA-IS is much lower than that of RA-SG and RA-VD. This is mainly due to the difference in volume shrinkage (Figure 3). RA-IS possesses the best pore structure for thermal insulation, along with the highest pore volume (1.875 cm^3^·g^−1^) and specific surface area (401 m^2^·g^−1^). RA-VD exhibits the smallest specific surface area and pore volume.

### 2.3. Hydrophobicity

**Figure 4:**
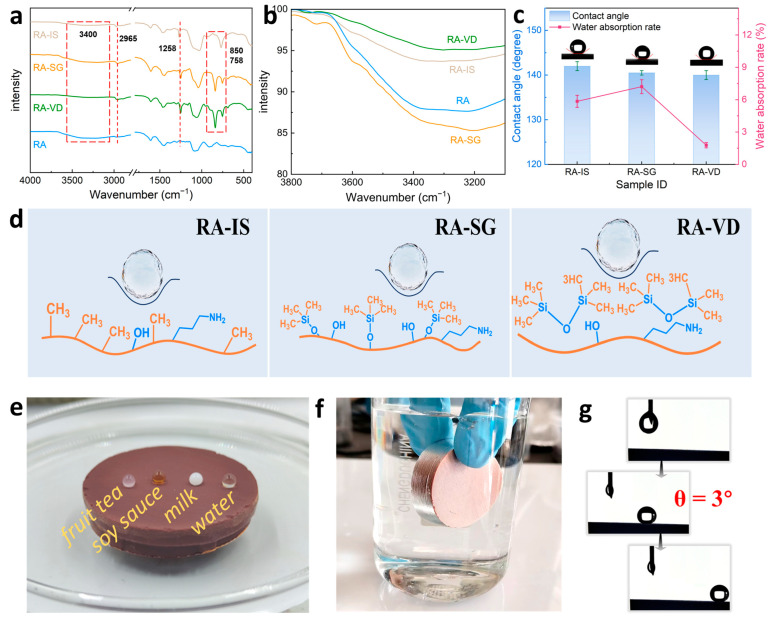
(**a,b**) FTIR spectra of RA, RA-IS, RA-SG, and RA-VD; (**c**) water contact angles and liquid water absorption rates of RA-IS, RA-SG, and RA-VD; (**d**) water-repellent mechanisms of RA-IS, RA-SG, and RA-VD; (**e**) photograph of a floating RA-IS monolith with different liquid droplets on its surface; (**f**) demonstration of hydrophobicity in liquid water; (**g**) sliding of water droplets on RA-IS.

The Fourier transform infrared (FTIR) spectra of RA-IS, RA-SG, and RA-VD are shown in Figure 4a,b. The bands at 1258 cm^−1^ are ascribed to the symmetric bending vibrations of C–H (Si–CH_3_) [50]. The peaks at 758 cm^−1^ are attributed to the stretching vibration of Si-C belonging to Si-CH_3_. The bands at 850 cm^−1^ are assigned to the stretching vibration of Si-C, which is derived from Si-CH_2_-CH_2_-CH_2_-NH_2_ and Si-CH_3_ [51]. The Si-C stretching of Si-CH_2_-CH_2_-CH_2_-NH_2_ and Si-CH_3_ around 850 cm^−1^ is weak, as is the Si-C stretching of Si-CH3 loaded by in situ polymerization [52]. However, the Si-C stretching of Si-CH_3_ loaded by the surface grafting of HMDS is stronger and more easily observed [53]. The methyl species in RA-SG and RA-VD are loaded by the surface grafting of HMDS, while Si-CH_2_-CH_2_-CH_2_-NH_2_ and Si-CH_3_ loaded by in situ polymerization are observed in RA-IS. Therefore, the band at 850 cm^−1^ for RA-IS is much weaker than that for RA-SG and RA-VD. The bands around 3400 cm^−1^ correspond to the stretching vibrations of O–H, which are derived from adsorbed water. For RA-SG, RA-IS, and RA-VD, the signal becomes weaker and weaker (Figure 4b).

Water contact angles and liquid water absorption rates of RA-IS, RA-SG, and RA-VD are shown in Figure 4c. With similar water contact angles ranging from 140 to 142°, the water absorption rate of RA-VD (1.8%) is much lower than that of RA-IS (5.8%) and RA-SG (7.2%), consistent with the results obtained from FTIR spectra. The difference in water absorption rate is explained by the water-repellent mechanism (Figure 4d). According to the formulation of hydrophobic RF/SiO_2_ aerogels (Table S4), the amounts of hydrophilic aminopropyl groups in RA-VD and RA-SG are the same. However, the aminopropyl sites in RA-VD can be covered by long-chain HMDS species completely, which can repel liquid water efficiently. For RA-SG, aminopropyl can be exposed on the surface, although methyl species are grafted on surface hydroxyl sites. For RA-IS, surface methyl groups are dominant, as the MTMS/APTES molar ratio is 4. However, there are a small number of hydrophilic aminopropyl groups available for water absorption. RA-IS is selected for the demonstration of broad-spectrum hydrophobicity. A monolithic RA-IS sample supports the beading of various liquids on its surface, including fruit tea, soy sauce, milk, and purified water (Figure 4e). During water immersion, a silvery-white air film forms on the surface of RA-IS, fully demonstrating its high hydrophobic performance (Figure 4f). When RA-IS is tilted at 3°, water droplets roll off a 10 mm slope within 0.3 s (Figure 4g), indicating the uniform distribution of surface methyl groups.

### 2.4. Weather Resistance

**Figure 5:**
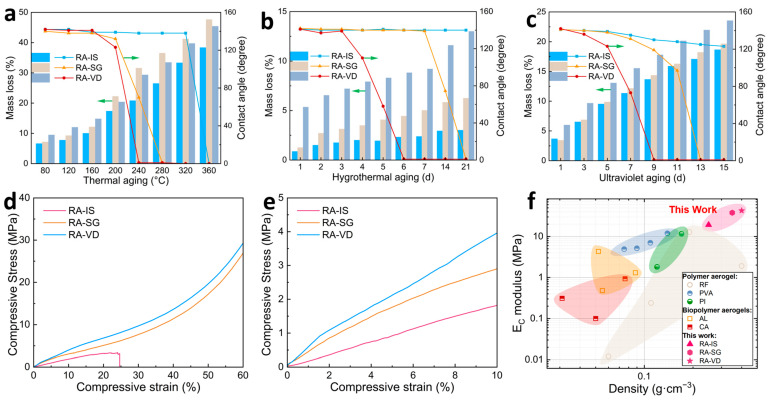
(**a**) Mass losses and contact angles of RA-IS, RA-SG, and RA-VD after thermal aging at different temperatures for 1 h; (**b**) mass losses and contact angles of RA-IS, RA-SG, and RA-VD after hygrothermal aging at 85 °C and 85% relative humidity; (**c**) mass losses and contact angles of RA-IS, RA-SG, and RA-VD after UV irradiation with a power of 40 W and a distance of 15 cm; (**d**) compressive stress–strain behavior under 60% deformation; (**e**) Compressive stress–strain behavior under 10% deformation; (**f**) comparison of aerogel densities and Young’s modulus with those of other polymer and bio-based aerogels [55,56,57,58,59,60,61,62,63,64,65,66,67,68].

The stability of hydrophobicity is crucial for building insulation materials. Without hydrophobicity, porous structures can be destroyed by water, and thermal insulation performance may decline rapidly. Therefore, building insulation materials should have excellent weather resistance under complex outdoor conditions. The stability of hydrophobic RF/SiO_2_ aerogels is evaluated under thermal, hygrothermal, and ultraviolet (UV) aging conditions. For thermal aging at different temperatures for 1 h (Figure 5a), RA-IS maintains high hydrophobicity at elevated temperatures up to 320 °C, while RA-SG and RA-VD show a significant decline in hydrophobicity above 200 °C. Simultaneously, the mass loss of RA-IS is much smaller than that of RA-SG and RA-VD under high temperatures. For hygrothermal aging at 85 °C and 85% relative humidity (Figure 5b), the hydrophobicity of RA-IS hardly changes within 21 d, while there is minor mass loss below 3%. RA-SG loses hydrophobicity after aging for 14 d, along with moderate mass loss of 6.3%. RA-VD loses hydrophobicity after aging for 5 d, along with remarkable mass loss of up to 13%. For UV irradiation with a power of 40 W and a distance of 15 cm (Figure 5c), RA-IS shows the best durability, preserving hydrophobicity within 15 d along with a mass loss of 18.6%. The hydrophobicity of RA-VD degrades rapidly within 7 d, exhibiting high mass loss of up to 23.5%. The mass loss of RA-SG is close to that of RA-IS; however, its hydrophobicity disappears at 13 d. Overall, RA-IS demonstrates the best stability in terms of hydrophobicity under different aging conditions, while RA-VD exhibits the worst stability. The difference in the stability of hydrophobicity reflects the stability of methyl species loaded in the hydrophobic RF/SiO_2_ aerogels via different modification methods. As mentioned earlier (Figure 1b), the methyl species loaded by covalent bond in RA-IS and RA-SG are more stable than those loaded by physical adsorption in RA-VD. Furthermore, the methyl groups fixed in the matrix of RA-IS are more robust than those grafted onto the surface of RA-SG. The results highlight the critical role of hybridization in the framework via covalent bonding in stabilizing methyl groups.

### 2.5. Mechanical Properties

The mechanical properties of hydrophobic RF/SiO_2_ aerogels were evaluated by uniaxial compression. The stress–strain curves of RA-IS, RA-SG, and RA-VD are shown in Figure 5d,e. The compressive stress–strain curves of the samples exhibit an initial quasi-linear region followed by progressive nonlinear deformation and densification, which is characteristic of polymer-based aerogels and indicates that the samples behave as solid-like viscoelastic materials rather than ideally elastic solids. This behavior reflects time-dependent deformation under load, similar to the viscoelastic response reported for polymer nanocomposites with strong polymer–filler interactions [54]. These results support the classification of the synthesized RF/SiO_2_ aerogels as viscoelastic solids under the quasi-static loading conditions used in this work. RA-VD showed the highest strength and stiffness, reaching 3.9 MPa at 10% strain and 28.8 MPa at 60% strain, with a Young’s modulus of 42.8 MPa. This superior performance is attributed to its densified network and higher carbon content: vapor-phase HMDS treatment not only introduces hydrophobic groups but also partially fills the framework, reduces porosity, and enhances mechanical interlocking between adjacent nanoparticles, thereby preventing collapse under large compressive strains. High-strength RA-VD supports a high weight, as demonstrated in Figure S1. Although there is 13% deformation, the structure remains intact, and the morphology is not damaged. RA-SG attains 2.9 MPa at 10% strain and 26.6 MPa at 60% strain, with a Young’s modulus of 38 MPa. Although less dense than RA-VD, covalent Si–O–Si linkages formed during chemical grafting reinforce contacts and improve stress transfer, yielding a balanced strength–porosity profile. In contrast, RA-IS fractures at 25% strain, with a peak strength of 3.3 MPa and Young’s modulus of 19.2 MPa, consistent with a brittle, loosely cross-linked hybrid network. Excess silica disrupts matrix continuity, generating mesopores and stress concentrators that accelerate crack propagation. The strengths of hydrophobic RF/SiO_2_ aerogels and their state-of-the-art counterparts are shown in Figure 5f. The strengths of RA-IS, RA-SG, and RA-VD are much higher than those of phenolic aerogel (RF), polyvinyl alcohol aerogel (PVA), polyimide aerogel (PI), alginate aerogel (AL), and cellulose aerogel (CA) [55,56,57,58,59,60,61,62,63,64,65,66,67,68].

### 2.6. Thermal Performace

**Figure 6:**
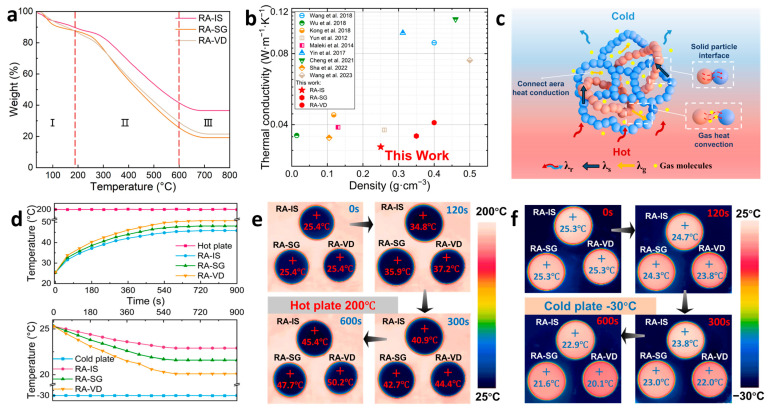
(**a**) TG curves of RA-IS, RA-SG, and RA-VD; (**b**) thermal conductivities of RA-IS, RA-SG, RA-VD and other phenolic/silica hybrid aerogels [23,24,44,69,70,71,72,73,74]; (**c**) thermal transfer mechanism of aerogels; (**d**) back temperatures of RA-IS, RA-SG, and RA-VD on hot and cold plates (the thickness of the samples is 1 cm); (**e**) thermal imaging changes on A 200 °C hot plate; (**f**) thermal imaging changes on a −30 °C cold plate.

TG curves of RA-IS, RA-SG, and RA-VD are shown in Figure 6a. All samples exhibit a three-stage mass loss profile. Stage I (50–200 °C) is associated with the removal of physiosorbed water and residual ethanol or other low-boiling species trapped in the nanopores, resulting in a noticeable but limited mass loss. This behavior is typical of high-surface-area aerogels, even when they are rendered hydrophobic, due to the moisture adsorption during storage. Stage II (200–600 °C) corresponds to the decomposition of the RF/SiO_2_ network and the partial cleavage of organic–inorganic linkages. The RA-IS shows a markedly higher onset temperature than RA-SG and RA-VD (Figure S2), consistent with the formation of a protective Si–O–Si network. Stage III (600–800 °C) involves the further carbonization of organics and the decomposition of silicon-containing moieties, leaving an inorganic, silica-rich residue. RA-IS gives the highest residual mass, reflecting its denser siliceous framework and improved thermal resistance. The thermal conductivities of hydrophobic RF/SiO_2_ aerogels and other reported phenolic/inorganic hybrid aerogels are shown in Figure 6b. The thermal conductivities at 25 °C are 32.2, 35.8, and 40.8 mW·m^−1^·K^−1^ for RA-IS, RA-SG, and RA-VD, respectively. Compared with RA-SG, RA-VD, and other phenolic/silica hybrid aerogels, RA-IS has much lower thermal conductivity at 25 °C [23,24,44,69,70,71,72,73,74]. The low thermal conductivity of RA-IS is attributed to its low bulk density, high specific surface area and high nanopore volume. This can be explained by the thermal transfer mechanism. As shown in Figure 6c, the heat transfer is mainly contributed by solid-phase heat transfer (λ_s_), gas-phase heat transfer (λ_g_), and thermal radiation (λ_r_) [75,76,77]. Under low temperatures, the thermal radiation is ignorable. Solid-phase heat transfer can be suppressed efficiently by increasing the connect area of nanoparticles constructedthe framework, which is achieved by reducing the density and increasing the specific surface area. Gas-phase heat transfer can be suppressed by increasing the nanopore volume as the heat convection of gas is restricted in small pores, leaving weak thermal transfer by the heat conduction of gas. Consequently, RA-IS shows the lowest overall thermal conductivity among the three samples.

The practical thermal insulation performances under cold and hot environments in terms of building insulation are evaluated by fixing the aerogels on the 200 and −30 °C plates to measure the back temperature. The back temperatures of the RA-IS, RA-SG, and RA-VD samples with a thickness of 1 cm on the 200 °C and −30 °C plates are shown in Figure 6d. RA-IS exhibits the best thermal insulation performance, along with the largest temperature differences. RA-VD has the worst insulation performances, exhibiting 4.8 and 2.8 °C lower temperature differences on the 200 and −30 °C plates, respectively. The results can be explained by the thermal transfer mechanism mentioned earlier. Infrared thermography (Figure 6e,f) further shows that all aerogels effectively block heat flow, with RA-IS consistently delivering the largest gradient under both hot and cold conditions.

### 2.7. Flame Resistance

**Figure 7:**
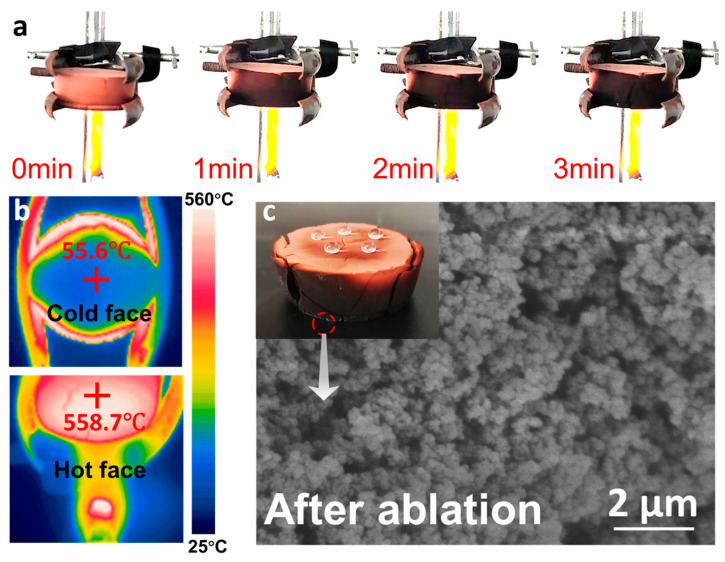
(**a**) Flame resistance of RA-IS on an alcohol flame (the thickness of the samples is 2 cm); (**b**) infrared images of the cold and hot surfaces of RA-IS after burning on the alcohol flame for 3 min; (**c**) SEM images of RA-IS after burning on the alcohol flame for 3 min.

The RA-IS aerogel, identified as the most thermally resistant formulation, was subjected to an alcohol burner flame ablation test using a 2 cm thick specimen. After continuous flame exposure for 3 min (Figure 7a), the sample maintained structural integrity without visible collapse or macroscopic deformation, demonstrating robust thermal shielding. Infrared thermography (Figure 7b) revealed a steep through-thickness gradient: the flame-facing surface rapidly reached high temperatures, whereas the back surface remained near ambient temperature, yielding a temperature drop exceeding 500 °C across 2 cm. SEM observations (Figure 7c) show partial sintering and localized shrinkage after exposure, yet the porous skeleton remains continuous, evidencing the high-temperature structural stability of the RA-IS network. Consistently, static water contact angle measurements on the non-impinged face indicate that hydrophobicity is largely retained, underscoring the durability of the hydrophobic modification under flame conditions.

## 3. Conclusions

Hydrophobic RF/SiO_2_ hybrid aerogels were prepared from a hydrophilic RF/SiO_2_ precursor by three methyl modification routes: in situ polymerization (RA-IS), surface grafting (RA-SG) and vapor deposition (RA-VD). All samples are highly hydrophobic, with water contact angles around 140°. In RA-IS, the methyl groups are covalently built into the framework, whereas in RA-SG they are mainly grafted onto the surface and in RA-VD they are largely physically adsorbed. Therefore, RA-IS demonstrates the best stability in terms of hydrophobicity under thermal, hygrothermal, and ultraviolet aging conditions. Owing to less pore blocking and shrinkage, RA-IS shows the lowest bulk density, the highest specific surface area and pore volume, and thus the lowest thermal conductivity (32.2 mW·m^−1^·K^−1^ at 25 °C). Its compressive strength (3.3 MPa) and Young’s modulus (19.2 MPa) are higher than those of most reported polymer and bio-based aerogels, although they are smaller than those of the denser RA-SG and RA-VD. RA-IS is incombustible and retains its microstructure on an alcohol flame. Overall, this study clarifies how the methyl modification route controls hydrophobicity, structure and performance and provides guidance for designing RF/SiO_2_ aerogels for durable, fire-safe building insulation.

## 4. Materials and Methods

### 4.1. Materials

Ethanol (99.7%, CAS 64-17-5) was provided by Wuxi Yasheng Chemical Co., Ltd., Wuxi, China. R (AR, CAS 108-46-3) was provided by Sinopharm Chemical Reagent Co., Ltd., Shanghai, China. F (37 wt % aqueous solution, AR, CAS 50-00-0) was provided by Xilong Science Co., Ltd., Shantou, China. APTES (AR, CAS 919-30-2), MTMS (AR, CAS 1185-55-3) and HMDS (AR, CAS 999-97-3) were purchased from Shanghai Aladdin Biochemical Technology Co., Ltd., Shanghai, China.

### 4.2. Synthesis

The composition of all sample materials is shown in Table S4.

#### 4.2.1. Synthesis of RA

Under room temperature, R (11 g), F (15 mL), APTES (12 mL), and ethanol (145 mL) were mixed to form a homogeneous solution under stirring. The resulting solution was sealed in a plastic mold. Then, gelation and aging occurred 50 °C for 24 h, producing a wet gel. The wet gel was immersed in ethanol to conduct solvent exchange at 50 °C for 3 d to obtain an alcogel, and the ethanol was refreshed each day. The alcogel underwent supercritical CO_2_ drying at 10 MPa and 50 °C in an autoclave with a CO_2_ flow rate of 10 L·min^−1^ to obtain RA.

#### 4.2.2. Synthesis of RA-IS

The synthesis procedures for RA-IS were the same as those for RA. To achieve hydrophobicity, MTMS was used as co-precursor of silica. The ethanol amount was increased to obtain the same theoretical density as that of RA. The theoretical density calculation formula is shown in Section S1. For a given target composition (fixed molar ratios of R, F, APTES and MTMS), the total amount of solid precursors was kept constant. When additional MTMS was introduced (e.g., in RA-IS) or when the ratio between organic and inorganic precursors was modified, the volume of ethanol in the sol was increased such that the initial solid volume fraction (and hence the theoretical density of the resulting solid framework) remained equal to that of the reference RA sample.

#### 4.2.3. Synthesis of RA-SG

The alcogel of RA was immersed in an HMDS/ethanol solution at 50 °C for 2 d to conduct surface grafting. The resulting solvent exchange and supercritical CO_2_ drying procedures were the same as those for RA.

#### 4.2.4. Synthesis of RA-VD

To conduct vapor deposition of HMDS, RA was placed in a sealed container with HMDS on the bottom. The container was vacuumized to form efficient HMDS vapor. The vacuum degree, temperature of the container and treatment time for RA varied, with details provided in Table S3. The resulting RA-VD samples were treated in an air blast drying oven for 1 h at 50 °C.

### 4.3. Characterization Methods

The bulk density of the aerogels was calculated from their mass and geometric volume, which were obtained using an analytical balance and a vernier caliper, respectively. Each reported density corresponds to the average of five measurements performed on different subsamples. The microstructures were examined by SEM (Regulus 8100, Hitachi, Ibaraki, Japan). Thermal stability was investigated by thermogravimetric analysis (TGA 550, TA Instruments, New Castle, DE, USA) under nitrogen, with the temperature ramped from 25 °C to 800 °C at a heating rate of 10 °C·min^−1^. The chemical structure was characterized by Fourier transform infrared spectroscopy (FTIR, IRSpirit-T, Shimadzu, Kyoto, Japan) over the wavenumber range of 4000–400 cm^−1^. Nitrogen adsorption–desorption measurements were carried out using a BET surface area and pore size analyzer (BELSORP MINI X, MicrotracBEL, Osaka, Japan) to determine the specific surface area and pore structure. Surface hydrophobicity was evaluated from static water contact angle measurements using a contact angle meter (MIT-100S, Changzhou Sanfeng Instrument Technology Co., Ltd., Changzhou, China). The thermal conductivity at 25 °C was measured with a plate-type thermal conductivity tester (DRPL-IV, Xiangtan Xiangyi Instrument Co., Ltd., Xiangtan, China). Thermal protection performance was assessed by placing the specimens on a heating stage and monitoring the temperature distribution with an infrared thermal imaging camera (UTi 260B, UNI-T, Dongguan, China). Uniaxial compressive properties were obtained on a universal testing machine (DECA FL 4204 GL, Meters Industrial Systems Co., Ltd., Shanghai, China) at a loading rate of 1 mm·min^−1^. Environmental durability, including resistance to high temperatures, humidity aging and UV aging, was evaluated using a high-temperature muffle furnace (KJ-XB14, Luoyang Keju Luoye Co., Ltd., Luoyang, China), a humidity chamber (BPS-100CH, Shanghai Yiheng Scientific Instruments Co., Ltd., Shanghai, China) and an ultraviolet aging chamber (UVA-340, Gongyouji Industrial Technology Co., Ltd., Shenzhen, China), respectively.

## Figures and Tables

**Table 1:** Pore structure data of hydrophobic RF/SiO_2_ aerogels.

Sample	Theoretical Density (g·cm^−3^)	Bulk Density (g·cm^−3^)	Specific Surface Area (m^2^·g^−1^)	Pore Volume (cm^3^·g^−1^)
RA-IS	0.12	0.25 ± 0.01	401	1.875
RA-SG	0.12 *	0.35 ± 0.02	377	1.869
RA-VD	0.12 *	0.40 ± 0.02	310	1.450

* The theoretical densities are defined based on RA.

## Data Availability

The data is contained within the article or Supplementary Materials.

## Supplementary Materials

The following supporting information can be downloaded at https://www.mdpi.com/article/10.3390/gels12010004/s1, Figure S1: (a) Adult male standing on RA-VD; (b) Compressive deformation before and after the experiment; Figure S2: DSC of RA-IS, RA-SG, and RA-VD; Table S1: Water contact angles of the RA-IS samples with different R/MTMS molar ratios; Table S2: Water contact angles of the RA-SG samples with different molar fractions in the HMDS/EtOH solution; Table S3: Water contact angles of the RA-GD samples with different modification environments; Table S4: Dosages of RA, RA-IS, RA-SG and RA-VD. Section S1: Theoretical density and bulk density calculation methods.
