# Supplementary material for: Hydrophobic Phenolic/Silica Hybrid Aerogels for Thermal Insulation: Effect of Methyl Modification Method
Source: Gels. 2025 Dec 20;12(1):4. doi: 10.3390/gels12010004 (PMC12841154; doi:10.3390/gels12010004)
Supplement: Supplementary file 1 [file gels-12-00004-s001.zip › gels-4025866-supplementary.pdf]

Supporting Information

Hydrophobic Phenolic/Silica Hybrid Aerogels for Thermal Insulation: Effect of Methyl Modification Method

Mengcheng Nie [1], Yong Kong [1,2,*], Zhixin Wang [1], Fuhao Xu [1], Jiantao Zhou [1] and Xiaodong Shen [1,2]

[1] College of Materials Science and Engineering, Nanjing Tech University, Nanjing 211816, China
[2] Jiangsu Collaborative Innovation Center for Advanced Inorganic Function Composites, Nanjing 211816, China
* Correspondence: ykong@njtech.edu.cn

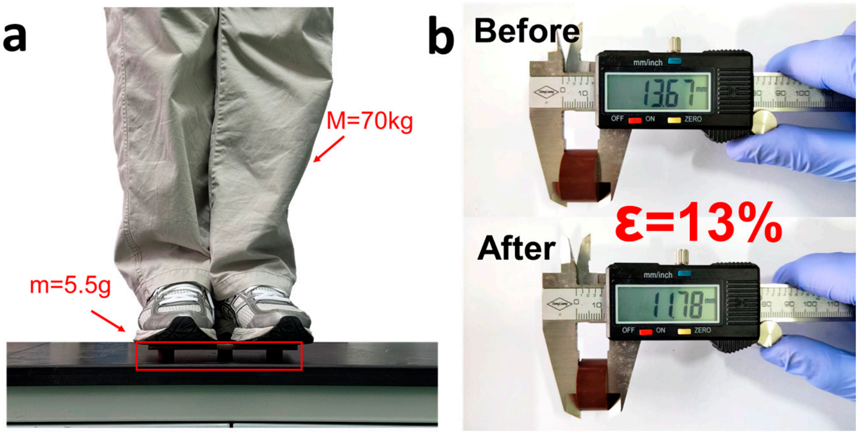

Figure S1. (a) Adult male standing on RA-VD; (b) Compressive deformation before and after the experiment.

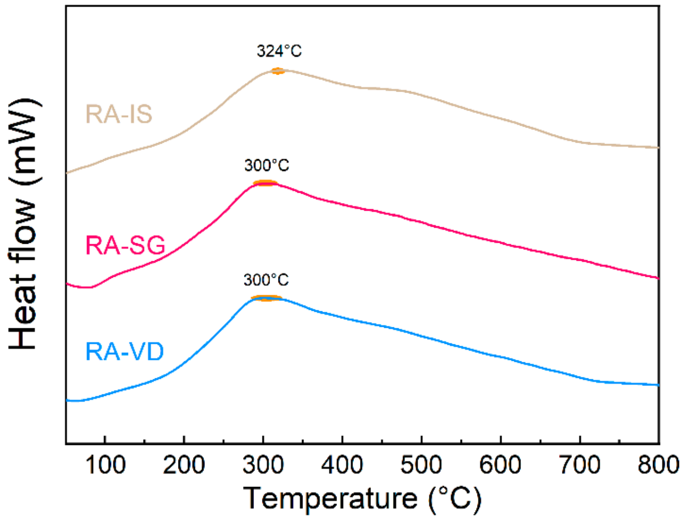

Figure S2. DSC of RA-IS, RA-SG, and RA-VD.

Table S1. Water contact angles of the RA-IS samples with different R/MTMS molar ratios.

R/MTMS molar ratio	Water contact angle (°)
0.5	0
1	57±4
1.5	121±3.2
2	142±1.2

Table S2. Water contact angles of the RA-SG samples with different molar fractions in the HMDS/EtOH solution.

HMDS molar fraction in HMDS/EtOH solution (%)	Water contact angle (°)
1	0
5	93±3
10	141±0.6
30	141±0.3

Table S3. Water contact angles of the RA-GD samples with different modification environments.

Temperature (°C)	Time (h)	Vacuum degree (MPa)	Water contact angle (°)
25	6	0.06	0
60	3	0.06	0
60	6	0.03	103±5
60	6	0.06	140±1.5
60	9	0.06	140±0.8
80	6	0.06	141±0.5
60	6	0.09	141±0.4

Table S4. Dosages of RA, RA-IS, RA-SG and RA-VD.

Sample	R (mol)	F (mol)	APTES (mol)	MTMS (mol)	EtOH (mol)
RA	0.1	0.2	0.05	0	2.5
RA-SG	0.1	0.2	0.05	0	2.5
RA-VD	0.1	0.2	0.05	0	2.5
RA-IS	0.1	0.2	0.05	0.2	3.7

Section S1. Theoretical density and bulk density calculation methods

The theoretical density of the solid framework (ρ_{th}) was estimated from the contribution of each solid phase derived from the precursors (phenolic resin from R/F and silica from APTES and MTMS), assuming complete reaction. It was calculated according to:

$$\rho_{th} = \frac{\sum_i m_i}{\sum_i \frac{m_i}{\rho_i}} \tag{S1}$$

Where m_i is the mass of solid component i, ρ_i is its true density. In this work, the solid components i correspond to the phenolic phase (from R/F) and the silica phase (from APTES and MTMS) in the dried hybrid aerogels.

The bulk density (ρ_{bulk}) of the aerogels was calculated from the measured mass and the geometric volume of the dried monoliths according to:

$$\rho_{bulk} = \frac{m}{V} \tag{S2}$$

Where m is the mass of the sample and V is its external geometrical volume determined from the measured dimensions.
